# Impact of hepatic steatosis on mortality, hepatocellular carcinoma, end-stage liver disease and HBsAg seroclearance in chronic hepatitis B: a United States cohort study

**DOI:** 10.3389/fimmu.2025.1566925

**Published:** 2025-04-02

**Authors:** George A. Yendewa, Abbinaya Elangovan, Temitope Olasehinde, Frank Mulindwa, Mackenzie G. Cater, Robert A. Salata, Jeffrey M. Jacobson

**Affiliations:** ^1^ Department of Medicine, Case Western Reserve University School of Medicine, Cleveland, OH, United States; ^2^ Division of Infectious Diseases and HIV Medicine, University Hospitals Cleveland Medical Center, Cleveland, OH, United States; ^3^ Department of Medicine, United Health Services Wilson Memorial Hospital, Johnson City, NY, United States

**Keywords:** steatotic liver disease, hepatitis B virus, mortality, hepatocellular carcinoma, cirrhosis, fibrosis, end-stage liver disease

## Abstract

**Background:**

Steatotic liver disease (SLD) is prevalent among individuals with chronic hepatitis B virus (CHB), yet its impact on clinical outcomes remains controversial.

**Methods:**

We used electronic health record data from 98 US healthcare-delivery systems to compare adults with (CHB-SLD) and without SLD (CHB-wo-SLD) from 2000 to 2024. We applied 1: 1 propensity score matching to balance cohorts by demographic and clinical characteristics. We further performed sensitivity analyses in the presence or absence of cirrhosis. We compared incidence rates (IR) and hazard ratios (HRs) of all-cause mortality, hepatocellular carcinoma (HCC), end-stage liver disease (ESLD) events, and detectable HBsAg and HBeAg as markers of seroclearance.

**Results:**

Among 124,932 individuals with CHB (12.43% CHB-SLD), there were 470,707 person-years of observations (median follow-up 2.95 years). Compared with CHB, individuals with CHB-SLD had a lower mortality risk (HR 0.44, 95% CI 0.40-0.48). Fibrosis risk was higher among those with CHB-SLD (vs CHB-wo-SLD) (HR 1.93, 95% CI 1.71-2.19); however, cirrhosis risk was comparable (HR 1.06, 95% CI 0.96-1.18) between cohorts, while HCC risk was lower in the CHB-SLD cohort (HR 0.83, 95% CI 0.70-0.96). The CHB-SLD cohort also had significantly reduced risks of ESLD events, including ascites, spontaneous bacterial peritonitis, variceal bleeding, hepatic encephalopathy, and hepatorenal syndrome (all p < 0.001). Additionally, detectable HBsAg and HBeAg IRs and HRs were lower among CHB-SLD compared to the CHB-wo-SLD cohort: 26.83 vs. 31.96 per 1,000 person-years (HR 0.80, 95% CI 0.73-0.87) and 8.52 vs. 11.36 per 1,000 person-years (HR 0.74, 95% CI 0.65-0.85), respectively. Sensitivity analyses stratified by cirrhosis status supported these findings.

**Conclusion:**

CHB-SLD status was associated with more favorable outcomes, highlighting the complexity of CHB and SLD interactions.

## 1 Introduction

Steatotic liver disease (SLD), characterized by intrahepatic fat accumulation exceeding 5% of gross liver weight, is an increasingly recognized histological feature in hepatitis B virus (HBV) infection (1, 2). Recent estimates suggest that the prevalence of SLD in patients with chronic HBV (CHB) ranges from 25% to 40% (2–6). The mechanisms underlying the pathogenesis of SLD in CHB infection have not been elucidated; nonetheless, studies have suggested that age, male sex, insulin resistance, the metabolic syndrome, and the presence of certain single nucleotide polymorphisms (e.g., IL28B rs12979860 C>T and PNPLA3 rs738409 GG) are associated with an increased risk of developing SLD (2–8). These factors may influence the progression of SLD through chronic inflammation and immune dysregulation, which drive alterations in host lipid pathways (2, 9).

Despite the substantially high rates of co-occurrence, the impact of SLD on the clinical outcomes of people living with CHB remains controversial, with studies reporting contradictory findings (2, 9). A recent systematic review and meta-analysis conducted by Mao et al. (10) observed that individuals with concurrent CHB and SLD had a higher risk of developing liver cirrhosis and hepatocellular carcinoma (HCC) but had a higher likelihood of achieving functional cure, i.e., seroclearance of hepatitis B surface antigen (HBsAg), compared with those without SLD. However, another systematic review and meta-analysis by Wong et al. (11) reported a lower incidence of HCC, cirrhosis, and mortality in CHB patients with SLD than in their counterparts without SLD. These discrepancies may stem from variability in definitions of steatotic liver disease (SLD) and study methodologies, including differences in the criteria used to diagnose SLD, the stage of chronic liver disease, and the populations studied. For instance, previous studies often focused on entities such as nonalcoholic fatty liver disease or alcoholic fatty liver disease separately. However, recent revisions of the definition of SLD by an international panel of experts (12) and the American Association for the Study of Liver Diseases (13) incorporated a more inclusive framework, accounting for a variety of etiologies. Additionally, co-infections such as human immunodeficiency virus (HIV) and hepatitis C virus (HCV), which are prevalent among people living with CHB due to shared risk factors, significantly influence the clinical course and outcomes of both CHB infection and SLD (14, 15) but may be inconsistently accounted for in study analyses. These factors limit the generalizability of prior findings and contribute to the uncertainty about the true clinical implications of SLD in CHB. Addressing these knowledge gaps is critical to updating our understanding and optimizing management strategies for patients with concurrent CHB and SLD.

In this multisite cohort study from the United States using an updated and more inclusive definition of SLD, we aimed to: 1) compare the characteristics of individuals with CHB and concurrent SLD versus those without SLD; 2) estimate and compare the incidence and risk of clinical outcomes between these groups, including all-cause mortality, fibrosis, cirrhosis, hepatocellular carcinoma (HCC), and end-stage liver disease (ESLD) events; and 3) evaluate HBsAg and hepatitis B e antigen (HBeAg) seroclearance as markers of disease prognosis.

## 2 Materials and methods

### 2.1 Data source

We utilized the TriNetX database to conduct a retrospective multisite cohort study of adults aged ≥18 years with confirmed CHB infection who received care across 98 US integrated healthcare-delivery systems from January 1, 2000, to November 20, 2024 (last date of TriNetX access). TriNetX is a global federated health research network that provides access to de-identified data from electronic health records (EHRs), including diagnoses, procedures, medications, and laboratory values. To ensure the privacy of protected health information, TriNetX excludes data on the geographic and institutional details of the participating healthcare delivery systems. A typical participating healthcare-delivery system generally comprises a major academic health center with main and satellite hospitals, specialized care services, and outpatient clinics.

### 2.2 Cohort selection, study definitions, and outcomes

We included all adult patients with CHB infection (International Classification of Diseases, 10^th^ Revision codes, ICD-10: B18.0, B18.1). We then stratified patients with CHB into two cohorts for analysis: 1) patients with CHB and SLD, that is, CHB-SLD (ICD-10 codes: B18.0, B18.1, K70.0, K75.81, or K76.0); and 2) patients with CHB without SLD (CHB-wo-SLD). We excluded all individuals with HIV, HCV, or prior organ transplants from all cohorts, as these conditions are known to significantly influence the progression and outcomes of both CHB and SLD. We then collected data on patient demographics (age at index, body mass index [BMI], sex, race, and ethnicity), baseline comorbidities (ischemic heart disease, hypertensive disease, heart failure, diabetes, overweight or obesity, chronic kidney diseases, chronic lower respiratory diseases, neoplasms), lifestyle-associated risk factors (nicotine dependence, alcohol-related disorders), and antiviral treatments (entecavir, tenofovir disoproxil, tenofovir alafenamide, lamivudine, and adefovir).

Furthermore, we collected baseline laboratory parameters (within 6 months of SLD diagnosis), including complete blood counts (leukocytes, hemoglobin, and platelet counts), renal function tests (serum creatinine, glomerular filtration rate [GFR]), coagulation parameters (prothrombin time, international normalized ratio [INR], activated partial thromboplastin time [APTT]), liver function tests (aspartate transaminase [AST], alanine aminotransferase [ALT], transaminase [GGT], total and direct bilirubin, lipid panel [total cholesterol, high-density lipoprotein [HDL], low-density lipoprotein [LDL], triglycerides], alpha-fetoprotein (AFP), and HBV DNA and HBeAg.

The primary outcomes of interest were the incidence rates (IRs) and hazard ratios (HRs) of all-cause mortality, HCC, fibrosis, cirrhosis, hepatic fibrosis, ESLD events (ascites, spontaneous bacterial peritonitis [SBP], variceal bleeding, hepatic encephalopathy, and hepatorenal syndrome), and detectable serum HBsAg and HBeAg levels for seroclearance assessment. We further performed two sensitivity analyses to account for potential confounding from liver disease severity, as stage of liver disease may independently influence outcomes. Specifically, we compared outcomes in individuals with: 1) CHB-SLD and cirrhosis versus those with CHB-wo-SLD and cirrhosis to evaluate the impact of SLD in advanced liver disease; and 2) CHB-SLD without cirrhosis versus those with CHB-wo-SLD without cirrhosis to isolate the effect of SLD in the absence of advanced liver disease. A full description of the study definitions and variables used to query the TriNetX database and their corresponding ICD-10 codes is provided in **Supplementary Table 1**.

### 2.3 Statistical analyses

We performed primary and sensitivity analyses using the TriNetX Advanced Analytics platform. We presented continuous variables as mean ± standard deviation or median (interquartile range [IQR]) and categorical variables as frequencies and percentages. To balance the cohorts, we applied 1:1 greedy nearest-neighbor propensity score matching, adjusting for age at index, sex, race or ethnicity, BMI, comorbidities, HBV antiviral treatments, HBV DNA levels, and HBeAg-positive status. We compared continuous variables using independent Student’s t-tests and categorical variables using the chi-square test. For each individual, follow-up began at the index event—defined as the first documentation of CHB-SLD or CHB-wo-SLD diagnosis—and continued until an outcome, loss to follow-up, 20 years post-index, or censoring at the last date of TriNetX database access (November 20, 2024), whichever occurred first. We calculated the IRs of study outcomes (cases per 1,000 person-years) and their corresponding 95% confidence intervals (CIs). Using the Kaplan-Meier method, we estimated survival probabilities for study outcomes and compared their curves using the log-rank test. To assess HBsAg and HBeAg seroclearance rates, we derived the event probability as 100% minus the estimated survival probability, representing the cumulative incidence of seroclearance. We employed Cox proportional hazards models to compare time-to-event rates during the follow-up period and generated HRs with their corresponding 95% CIs. We evaluated the proportional hazards assumption using Schoenfeld residuals and set statistical significance at p < 0.05.

### 2.4 Patient consent statement

The study was approved by the IRB at the Case Western Reserve University/University Hospital Cleveland Medical Center. TriNetX received a waiver from the WCG IRB Connexus. Written informed consent was not required, as data from the TriNetX system safeguards patient privacy by reporting de-identified data.

## 3 Results

### 3.1 Baseline characteristics

A total of 124,932 individuals with CHB from 98 healthcare-delivery systems across the United States were included, of whom 15,532 (12.43%, 95% CI 12.00-12.86%) had SLD (**Table 1**). Before matching, the CHB-SLD and CHB-wo-SLD cohorts provided 470,707 person-years of observations with a mean follow-up of 3.82 ± 3.44 years and a median follow-up of 2.95 years (IQR 2.95-4.82). After matching, the cohorts contributed 97,259 person-years, with a mean follow-up of 3.82 ± 3.68 years and a median follow-up of 2.84 years (IQR 2.84-4.75).

**Table 1:** Comparison of baseline characteristics of CHB-SLD and CHB-no-SLD cohorts before and after propensity score matching.

Variables	Before Matching			After Matching		
	CHB-SLD	CHB-wo-SLD	p-Value	CHB-SLD	CHB-wo-SLD	p-Value
Total	15,532	109,400		12,730	12,730	
Mean (± SD) follow-up (years)	3.82 ± 3.44	3.76 ± 3.94		3.76 ± 3.46	3.88 ± 3.90	
Median (IQR) follow-up (years)	2.95 (2.95-4.82)	2.46 (2.46-5.16)		2.84 (2.84-4.75)	2.61 (2.61-5.33)	
Age at Index (years)^a^	53.8 ± 13.1	51.4 ± 15.6	<0.001	53.4 ± 13.1	53.9 ± 15.0	0.002
Gender						
Male	8,848 (57.3%)	56,711 (52.8%)	<0.001	7,338 (57.6%)	7,599 (59.7%)	0.001
Female	6,451 (41.8%)	50,066 (46.6%)	<0.001	5,278 (41.5%)	5,006 (39.3%)	0.001
Body mass index (kg/m^2^)^a^	28.6 ± 6.5	25.9 ± 5.8	<0.001	28.4 ± 6.4	26.9 ± 6.4	<0.001
Race or ethnicity						
Asian	7,557 (48.9%)	42,642 (39.7%)	<0.001	6,040 (47.4%)	6,024 (47.3%)	0.841
White	2,809 (18.2%)	13,729 (12.8%)	<0.001	2,212 (17.4%)	2,293 (18.0%)	0.183
Unknown Race	2,784 (18.0%)	34,827 (32.4%)	<0.001	2,603 (20.4%)	2,480 (19.5%)	0.054
Black or African American	1,382 (8.9%)	11,291 (10.5%)	<0.001	1,188 (9.3%)	1,189 (9.3%)	0.983
Hispanic or Latino	534 (3.5%)	2,125 (2.0%)	<0.001	402 (3.2%)	413 (3.2%)	0.695
Native Hawaiian or Other Pacific Islander	358 (2.3%)	1,177 (1.1%)	<0.001	231 (1.8%)	266 (2.1%)	0.113
Comorbidities						
Hypertensive diseases	5,907 (38.2%)	20,004 (18.6%)	<0.001	4,381 (34.4%)	4,483 (35.2%)	0.180
Ischemic heart diseases	1,781 (11.5%)	6,862 (6.4%)	<0.001	1,349 (10.6%)	1,423 (11.2%)	0.137
Heart failure	697 (4.5%)	2,916 (2.7%)	<0.001	557 (4.4%)	577 (4.5%)	0.543
Diabetes mellitus	3,475 (22.5%)	10,971 (10.2%)	<0.001	2,519 (19.8%)	2,559 (20.1%)	0.530
Overweight and obesity	2,612 (16.9%)	3,606 (3.4%)	<0.001	1,512 (11.9%)	1,503 (11.8%)	0.861
Chronic lower respiratory diseases	2,166 (14.0%)	7,516 (7.0%)	<0.001	1,629 (12.8%)	1,648 (12.9%)	0.722
Chronic kidney disease	1,225 (7.9%)	5,730 (5.3%)	<0.001	967 (7.6%)	984 (7.7%)	0.689
Diseases of liver	7,672 (49.7%)	13,728 (12.8%)	<0.001	5,222 (41.0%)	5,303 (41.7%)	0.303
Neoplasms	5,267 (34.1%)	25,005 (23.3%)	<0.001	4,099 (32.2%)	4,206 (33.0%)	0.153
Lifestyle-associated risk factors						
Nicotine dependence	1,303 (8.4%)	4,099 (3.8%)	<0.001	1,012 (7.6%)	1,042 (7.9%)	0.491
Alcohol related disorders	728 (4.7%)	1,727 (1.6%)	<0.001	541 (4.1%)	549 (4.1%)	0.805
Treatments						
Entecavir	1,628 (10.5%)	3,200 (3.0%)	<0.001	1,157 (9.1%)	1,188 (9.3%)	0.502
Tenofovir disoproxil	1,569 (10.2%)	5,214 (4.9%)	<0.001	1,070 (8.4%)	1,087 (8.5%)	0.702
Tenofovir alafenamide	822 (5.3%)	1,986 (1.8%)	<0.001	510 (4.0%)	522 (4.1%)	0.703
Lamivudine	242 (1.6%)	935 (0.9%)	<0.001	183 (1.4%)	196 (1.5%)	0.501
Adefovir	141 (0.9%)	342 (0.3%)	<0.001	92 (0.7%)	94 (0.7%)	0.883

^a^Mean ± standard deviation.

CHB-SLD, chronic hepatitis B with steatotic liver disease; CHB-wo-SLD, chronic hepatitis B without steatotic liver disease; SD, standard deviation.

Before matching, individuals with CHB-SLD (vs. CHB-wo-SLD) were older, predominantly male, had higher mean BMI, and included more Asians and Whites. They also had a higher prevalence of comorbidities, including ischemic heart disease, hypertensive diseases, diabetes mellitus, diseases of the liver, chronic lower respiratory diseases, chronic kidney disease, and neoplasms, and were more likely to have nicotine dependence, alcohol-related disorders, and receive antiviral HBV treatments (all p < 0.001). After matching, cohorts were balanced in demographics, comorbidities, lifestyle risk factors, and HBV treatment history, with residual imbalances in age, sex and mean BMI.

### 3.2 Baseline laboratory parameters

At baseline (within 6 months of CHB or SLD diagnosis), individuals with CHB-SLD showed evidence of better-preserved laboratory parameters, while those with CHB-wo-SLD demonstrated markers of greater end-organ damage (**Table 2**). Specifically, individuals with CHB-SLD (vs. CHB-wo-SLD) had higher hemoglobin levels, platelet counts, and lower creatinine levels. Coagulation parameters, including PT and INR, were better preserved in CHB-SLD. Notably, individuals with CHB-SLD demonstrated better-preserved liver function and synthetic activity, characterized by lower levels of AST, ALT, GGT, total and direct bilirubin, as well as higher levels of total protein and albumin (all p < 0.001). However, LDL, triglycerides, and HDL levels were significantly higher in CHB-SLD, consistent with the metabolic profile of SLD. HBV DNA levels were also lower in individuals with CHB-SLD. Even after matching, individuals with CHB-SLD demonstrated a higher likelihood of abnormal laboratory findings and markers of end-organ damage compared to their CHB-wo-SLD counterparts.

**Table 2:** Comparison of laboratory parameters obtained at or within 3 months of diagnosis in CHB-SLD and CHB-no-SLD patients before and after propensity score matching.

Laboratory parameters	Before Matching			After Matching		
	CHB-SLD	CHB-wo-SLD	p-Value	CHB-SLD	CHB-wo-SLD	p-Value
Leukocytes (x 10^9^/L)^a^	6.7 ± 5.8 11,681 (66.8%)	7.0 ± 5.9 47,330 (40.2%)	<0.001	6.8 ± 6.3 7,127 (56.0%)	6.7 ± 4.8 7,189 (56.5%)	0.008
Hemoglobin (g/dL) ^a^	13.8 ± 2.0 10,701 (69.3%)	13.0 ± 2.2 47,911 (44.6%)	<0.001	13.8 ± 2.0 8,260 (64.9%)	13.1 ± 2.2 8,303 (65.2%)	<0.001
Platelets (x 10^9^/L) ^a^	224 ± 80 10,751 (69.6%)	223 ± 89 45,305 (42.2%)	0.199	226 ± 79 8,191 (64.3%)	212 ± 89 8,240 (64.7%)	<0.001
Creatinine (mg/dL) ^a^	1.0 ± 1.8 11,353 (73.5%)	1.1 ± 1.3 48,642 (45.3%)	<0.001	1.0 ± 2.0 8,753 (68.8%)	1.1 ± 1.1 8,836 (69.4%)	0.005
GFR (mL/min/1.73 m^2^)	86 ± 27 11,118 (72.0%)	85 ± 33 47,453 (44.2%)	<0.001	86 ± 27 8,556 (67.2%)	84 ± 32 8,620 (67.7%)	<0.001
Hemoglobin A1c (%)^a^	6.3 ± 1.4 6,033 (39.1%)	6.2 ± 1.5 20,916 (19.5%)	<0.001	6.3 ± 1.5 4,471 (35.1%)	6.3 ± 1.5 4,521 (35.5%)	0.450
Prothrombin time (seconds) ^a^	12.4 ± 3.0 7,221 (46.8%)	12.3 ± 3.6 24,949 (23.2%)	<0.001	12.3 ± 3.1 5,246 (41.2%)	12.9 ± 3.9 5,301 (41.6%)	<0.001
INR ^a^	1.1 ± 0.3 7,378 (47.8%)	1.1 ± 0.4 25,383 (23.6%)	<0.001	1.1 ± 0.3 5,382 (42.3%)	1.1 ± 0.4 5,439 (42.7%)	<0.001
ATTP (seconds) ^a^	30.5 ± 8.5 4,373 (28.3%)	30.0 ± 8.5 19,793 (18.4%)	<0.001	30.4 ± 8.7 3,461 (27.2%)	30.8 ± 9.7 3,487 (27.4%)	0.084
ALP (IU/L) ^a^	87 ± 60 10,399 (67.3%)	98 ± 92 37,140 (34.6%)	<0.001	87 ± 60 7,817 (61.4%)	101 ± 90 7,883 (61.9%)	<0.001
AST (IU/L) ^a^	42 ± 96 11,511 (74.5%)	46 ± 134 48,021 (44.7%)	<0.001	41 ± 90 8,884 (69.8%)	53 ± 140 9,011 (70.8%)	<0.001
ALT (IU/L) ^a^	49 ± 115 11,638 (75.4%)	52 ± 162 49,703 (46.3%)	<0.001	49 ± 104 9,002 (70.7%)	59 ± 184 9,103 (71.5%)	<0.001
GGT (IU/L) ^a^	80 ± 197 2,774 (18.0%)	101 ± 214 6,367 (5.9%)	<0.001	85 ± 216 1,750 (13.7%)	105 ± 198 1,697 (13.3%)	0.005
Total bilirubin (mg/dL) ^a^	0.8 ± 1.2 10,925 (70.7%)	0.9 ± 2.0 41,805 (38.9%)	<0.001	0.8 ± 1.2 8,318 (65.3%)	1.1 ± 2.5 8,432 (66.2%)	<0.001
Direct bilirubin (mg/dL) ^a^	0.4 ± 1.1 5,604 (36.3%)	0.5 ± 1.8 19,373 (18.0%)	<0.001	0.3 ± 1.0 4,163 (32.7%)	0.7 ± 2.3 4,240 (33.3%)	<0.001
Total protein (mg/dL) ^a^	7.3 ± 0.7 9,079 (58.8%)	7.1 ± 0.9 30,121 (28.0%)	<0.001	7.3 ± 0.7 6,694 (52.6%)	7.1 ± 1.1 6,773 (53.2%)	<0.001
Albumin (g/dL*)* ^a^	4.2 ± 0.6 10,179 (65.9%)	4.0 ± 0.6 37,168 (34.6%)	<0.001	4.2 ± 0.6 7,662 (60.2%)	3.9 ± 0.7 7,727 (60.7%)	<0.001
Total cholesterol (mg/dL) ^a^	180 ± 44 7,413 (48.0%)	178 ± 43 26,435 (24.6%)	<0.001	182 ± 43 5,555 (43.6%)	175 ± 46 5,605 (44.0%)	<0.001
HDL (mg/dL) ^a^	48 ± 16 7,178 (46.5%)	52 ± 18 23,683 (22.0%)	<0.001	49 ± 16 5,327 (41.8%)	50 ± 18 5,394 (42.4%)	<0.001
LDL (mg/dL) ^a^	105 ± 37 6,972 (45.1%)	103 ± 36 23,332 (21.7%)	<0.001	106 ± 36 5,166 (40.6%)	101 ± 37 5,217 (41.0%)	<0.001
Triglycerides (mg/dL) ^a^	151 ± 142 6,444 (41.7%)	122 ± 101 20,703 (19.3%)	<0.001	152 ± 147 4,718 (37.1%)	124 ± 91 4,760 (37.4%)	<0.001
AFP (ng/mL) ^a^	4.1 ± 5.2 1,922 (12.4%)	5.6 ± 10.0 2,528 (2.4%)	<0.001	3.9 ± 4.7 1,262 (9.9%)	5.6 ± 9.5 1,195 (9.4%)	<0.001
HBV DNA (IU/mL) ^a^	141329 ± 1071273 2,385 (15.4%)	226389 ± 1459179 3,150 (2.9%)	<0.001	171,188 ± 1,260,449 1,454 (11.4%)	274,144 ± 1,465,943 1,379 (10.8%)	0.045
HBeAg positive	3,144 (20.4%)	8,260 (7.7%)	<0.001	473 (3.7%)	482 (3.8%)	0.963

a Mean ± standard deviation.

*n(%)* represents the number of individuals (n) with available laboratory values and the percentage (%) of the total population within each category.

AFP, alpha fetoprotein; ALT, alanine transaminase; AST, aspartate transaminase; ATTP, activated partial thromboplastin time; CI, confidence interval; CHB-SLD, patients with chronic hepatitis B and steatotic liver disease; HBeAg, hepatitis B e antigen; GGT, gamma-glutamyl transferase; INR, international normalized ratio; GFR, glomerular filtration rate; HBeAg, hepatitis B e antigen; HDL, high density lipoprotein; LDL, low density lipoprotein.

### 3.3 Incidence and risk of clinical outcomes in primary analysis

**Figure 1:**
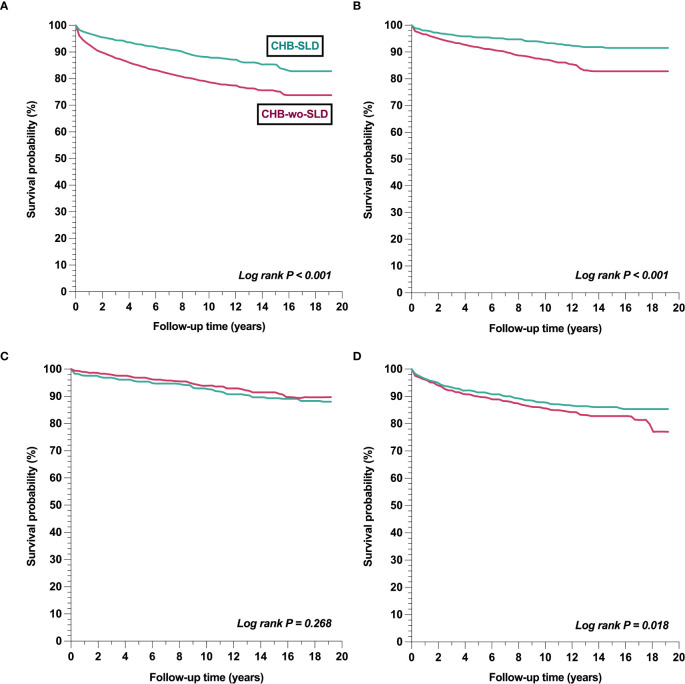
Comparison of survival probabilities of outcomes between CHB-SLD and CHB-wo-SLD **(a)** Mortality **(b)** Fibrosis **(c)** Cirrhosis **(d)** Hepatocellular carcinoma Footnote: CHB-SLD, patients with chronic hepatitis B and steatotic liver disease; CHB-wo-SLD, patients with chronic hepatitis B without steatotic liver disease.

In the matched cohort analysis (**Table 3**), individuals with CHB-SLD (vs. CHB-wo-SLD) had a lower incidence and risk of all-cause mortality (14.53 vs. 33.02 per 1,000 person-years; HR 0.44, 95% CI 0.40-0.48; p < 0.001). Despite having a higher risk of hepatic fibrosis (HR 1.93, 95% CI 1.71-2.19; p = 0.004), the risk of cirrhosis was similar between groups (HR 1.06, 95% CI 0.96-1.18; p = 0.268), whereas the incidence and risk of HCC was lower in individuals with CHB-SLD (6.11 vs. 7.50 per 1,000 person-years; HR 0.83, 95% CI 0.70-0.96; p = 0.018). Moreover, individuals with CHB-SLD had a lower risk of ESLD events, including ascites (HR 0.55, 95% CI 0.48-0.63; p < 0.001), spontaneous bacterial peritonitis (HR 0.36, 95% CI 0.25-0.53; p < 0.001), variceal bleeding (HR 0.64, 95% CI 0.54-0.76; p < 0.001), hepatic encephalopathy (HR 0.55, 95% CI 0.42-0.73; p < 0.001), and hepatorenal syndrome (HR 0.47, 95% CI 0.34-0.66; p < 0.001). **Figures 1A–D** illustrates the survival curves of all-cause mortality, fibrosis, cirrhosis, and HCC, respectively, for individuals with CHB-SLD compared to those with CHB-wo-SLD. Please refer to **Supplementary Table 2** for the incidence rates and hazard ratios of outcomes in the unmatched cohorts.

**Table 3:** Outcomes among CHB-SLD and CHB-no-SLD patients in primary analyses after propensity score matching.

Outcomes	Cohorts			IR (95% CI) (cases per 1,000 person-years)		HR (95% CI)	p-Value
	Overall	CHB-SLD	CHB-wo-SLD	CHB-SLD	CHB-wo-SLD		
General							
Mortality (all-cause)	25,336	690 (5.4%)	1,537 (12.1%)	14.53 (13.48-15.58)	33.02 (31.33-34.72)	0.44 (0.40-0.48)	<0.001
Hepatic							
Fibrosis	23,335	721 (6.2%)	384 (3.3%)	16.53 (15.35-17.72)	8.42 (7.58-9.26)	1.93 (1.71-2.19)	0.004
Cirrhosis	21,797	747 (6.7%)	685 (6.4%)	17.74 (16.49-19.00)	17.01 (15.76-18.25)	1.06 (0.96-1.18)	0.268
Hepatocellular carcinoma	23,914	280 (2.3%)	336 (2.9%)	6.11 (5.39-6.82)	7.50 (6.68-8.33)	0.83 (0.70-0.96)	0.018
End-stage Liver Disease							
Ascites	24,170	305 (2.5%)	541 (4.5%)	6.49 (5.73-7.24)	11.50 (10.53-12.48)	0.55 (0.48-0.63)	<0.001
Spontaneous bacterial peritonitis	25,346	39 (0.3%)	106 (0.8%)	0.85 (0.59-1.11)	2.23 (1.81-2.65)	0.36 (0.25-0.53)	<0.001
Variceal bleeding	24,669	220 (1.8%)	334 (2.7%)	4.71 (4.09-5.32)	8.83 (7.88-9.77)	0.64 (0.54-0.76)	<0.001
Hepatic encephalopathy	25,289	74 (0.6%)	133 (1.1%)	1.59 (1.23-1.96)	3.43 (2.84-4.01)	0.55 (0.42-0.73)	<0.001
Hepatorenal syndrome	25,352	50 (0.4%)	104 (0.8%)	1.08 (0.77-1.40)	2.17 (1.75-2.59)	0.47 (0.34-0.66)	<0.001
Virologic							
HBsAg seropositivity	24,834	927 (10.1%)	1,130 (12.4%)	26.83 (25.10-28.55)	31.96 (30.10-33.83)	0.80 (0.73-0.87)	<0.001
HBeAg seropositivity	23,397	375 (3.2%)	492 (4.2%)	8.52 (7.65-9.40)	11.36 (10.30-12.42)	0.74 (0.65-0.85)	<0.001

CI, confidence interval; CHB-SLD, patients with chronic hepatitis B and steatotic liver disease; CHB-wo-SLD, patients with chronic hepatitis B without steatotic liver disease; HBeAg, hepatitis B e antigen; HBsAg, hepatitis B surface antigen; HR, hazard ratio.

### 3.4 Incidence and risk of HBsAg and HBeAg seroclearance in primary analysis

**Figure 2:**
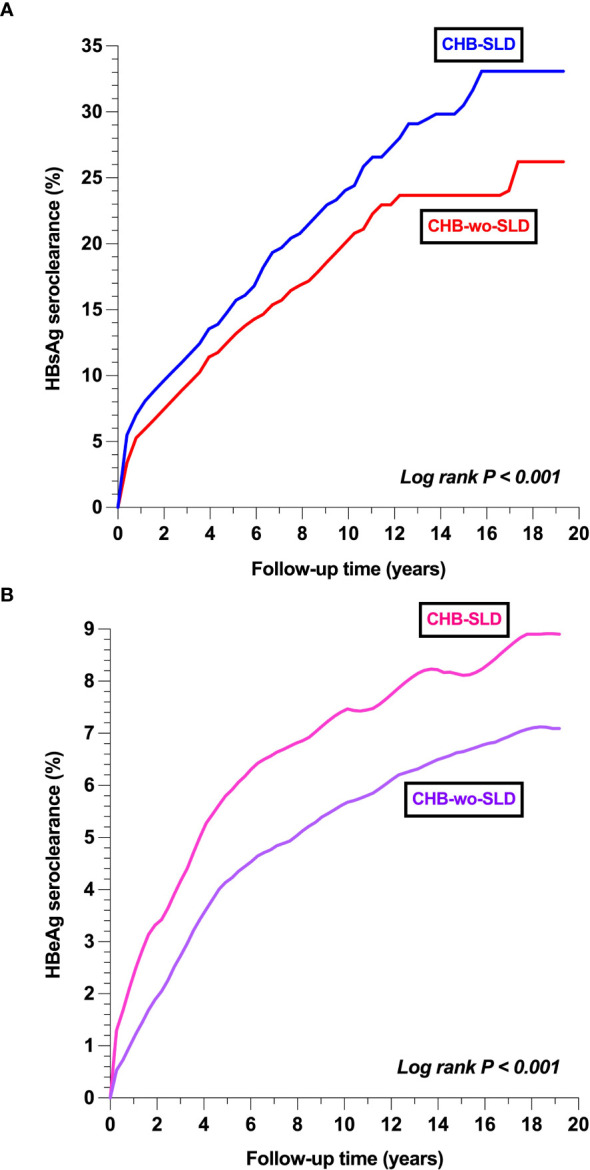
Comparison of event probabilities of virologic outcomes between CHB-SLD and CHB-wo-SLD **(a)** HBsAg seroclearance **(b)** HBeAg seroclearance Footnote: CHB-SLD, patients with chronic hepatitis B and steatotic liver disease; CHB-wo-SLD, patients with chronic hepatitis B without steatotic liver disease; HBeAg, hepatitis B e antigen; HBsAg, hepatitis B surface antigen.

The IR of detectable HBsAg was lower in the CHB-SLD group (vs. CHB-wo-SLD): 26.83 vs. 31.96 per 1,000 person-years (HR 0.80, 95% CI 0.73-0.87) (**Table 3**), indicating a higher rate of HBsAg seroclearance in individuals with CHB-SLD. Similarly, the IR of detectable HBeAg was lower in individuals with CHB-SLD (vs. CHB-wo-SLD): 8.52 vs. 11.36 per 1,000 person-years (HR 0.74, 95% CI 0.65-0.85), suggesting a higher rate of HBeAg seroclearance in the CHB-SLD group. **Figures 2A, B** illustrates the event probability curves for HBsAg and HBeAg seroclearance for individuals with CHB-SLD compared to those with CHB-wo-SLD, respectively. See **Supplementary Table 2** for IRs and HRs of detectable HBsAg and HBeAg in the sensitivity analysis of the unmatched cohorts.

### 3.5 Incidence and risk of outcomes in sensitivity analyses

The sensitivity analyses stratified by cirrhosis status largely aligned with the primary analysis while highlighting nuanced differences by cirrhosis status (**Table 4**). individuals with CHB-SLD had lower all-cause mortality compared with CHB-wo-SLD, regardless of cirrhosis (no cirrhosis: HR 0.47, 95% CI 0.41-0.53; p < 0.001; cirrhosis: HR 0.57, 95% CI 0.50-0.65; p < 0.001). Among those without cirrhosis, CHB-SLD was associated with higher fibrosis rates (HR 3.21, 95% CI 2.59-3.97; p < 0.001) but similar HCC risk (HR 1.21, 95% CI 0.88-1.43; p = 0.362). In contrast, CHB-SLD with cirrhosis had a lower HCC risk (HR 0.75, 95% CI 0.60-0.95; p = 0.017). ESLD event risks were comparable in the absence of cirrhosis, but individuals with CHB-SLD and cirrhosis had lower ESLD risks than CHB-wo-SLD. Detectable HBsAg and HBeAg rates were lower in CHB-SLD without cirrhosis (HBsAg: HR 0.76, 95% CI 0.69-0.84; p < 0.001; HBeAg: HR 0.64, 95% CI 0.55-0.75; p < 0.001), indicating higher seroclearance. In cirrhosis, HBsAg and HBeAg seroclearance rates were similar between groups. See **Supplementary Table 3** for IRs and HRs of the unmatched cohorts.

**Table 4:** Sensitivity analyses of CHB-SLD and CHB-wo-SLD cohorts with and without cirrhosis after propensity score matching.

Outcomes	No Cirrhosis					Cirrhosis				
	Overall	CHB-SLD	CHB- wo-SLD	HR (95% CI)	p-Value	Overall	CHB-SLD	CHB- wo-SLD	HR (95% CI)	p-Value
General										
Mortality (all-cause)	19,478	318 (3.3%)	701 (7.2%)	0.47 (0.41-0.53)	<0.001	4,918	369 (15.0%)	594 (24.2%)	0.57 (0.50-0.65)	<0.001
Hepatic										
Fibrosis	19,222	339 (3.6%)	114 (1.2%)	3.21 (2.59-3.97)	<0.001	2,916	117 (8.4%)	79 (5.2%)	1.66 (1.24-2.23)	0.004
Hepatocellular carcinoma	19,001	131 (1.4%)	126 (1.3%)	1.21 (0.88-1.43)	0.362	4,069	131 (6.0%)	149 (7.8%)	0.75 (0.60-0.95)	0.017
End-stage Liver Disease										
Ascites	19,229	93 (1.0%)	137 (1.4%)	0.70 (0.54-0.91)	0.007	3,927	188 (9.0%)	267 (14.6%)	0.58 (0.48-0.70)	<0.001
Spontaneous bacterial peritonitis	19,549	10 (0.1%)	10 (0.1%)	0.63 (0.21-1.92)	0.411	4,860	41 (1.7%)	76 (3.1%)	0.50 (0.34-0.74)	<0.001
Variceal bleeding	19,499	14 (0.1%)	12 (0.1%)	1.23 (0.57-2.67)	0.593	4,860	191 (8.9%)	220 (10.9%)	0.75 (0.61-0.91)	0.003
Hepatic encephalopathy	19,542	10 (0.1%)	11 (0.1%)	0.54 (0.20-1.47)	0.211	4,790	71 (2.9%)	105 (4.4%)	0.62 (0.46-0.83)	0.001
Hepatorenal syndrome	19,535	10 (0.1%)	10 (0.1%)	0.30 (0.10-1.09)	0.053	4,875	50 (2.0%)	65 (2.7%)	0.72 (0.50-1.04)	0.076
Virologic										
HBsAg	19,808	684 (9.2%)	895 (12.2%)	0.76 (0.69-0.84)	<0.001	4,652	206 (15.5%)	209 (15.8%)	0.94 (0.78-1.14)	0.557
HBeAg	18,085	260 (2.9%)	404 (4.5%)	0.64 (0.55-0.75)	<0.001	4,381	81 (3.7%)	89 (4.1%)	0.84 (0.62-1.13)	0.252

CI, confidence interval; CHB-SLD, patients with chronic hepatitis B and steatotic liver disease; CHB-wo-SLD, patients with chronic hepatitis B without steatotic liver disease; HBeAg, hepatitis B e antigen; HBsAg, hepatitis B surface antigen; HR, hazard ratio.

## 4 Discussion

In this multi-site cohort study, we analyzed real-time EHR data from 124,932 individuals with CHB across 98 U.S. healthcare systems between 2000 and 2024, including 15,532 (12.43%) with SLD. We found that individuals with CHB-SLD were older, predominantly male, and more frequently of Asian or White origin, reflecting the demographic characteristics of CHB in the US general population (16). Compared with those with CHB-wo-SLD, individuals with CHB-SLD had significantly lower risks of all-cause mortality and ESLD events, including ascites, spontaneous bacterial peritonitis, variceal bleeding, hepatic encephalopathy, and hepatorenal syndrome. Although CHB-SLD status was linked to an increased risk of hepatic fibrosis, cirrhosis risk were comparable between groups, while HCC risk was lower among those with CHB-SLD. Additionally, we observed higher rates of HBsAg and HBeAg seroclearance in CHB-SLD, suggesting a favorable prognosis in this group. Sensitivity analyses stratified by cirrhosis status supported these findings. Our observations align broadly with prior studies (10, 11), demonstrating the complex interaction between CHB and SLD, and offer useful insights for patient management and prognosis.

Among the clinical outcomes assessed, the association between SLD and the risk of HCC in individuals with CHB remains the most controversial. Individually, both CHB and SLD are well-established risk factors for HCC, suggesting that their coexistence could accelerate progression to liver-related complications, including HCC (9). However, as previously noted, some studies have reported a lower HCC risk in individuals with CHB-SLD, while others have found the opposite effect (10, 11). In this study, we sought to address methodological limitations, inconsistencies in inclusion criteria, and unaccounted confounding factors frequently observed in prior research that may have contributed to these divergent findings. To achieve this, we employed a propensity score-matching approach to balance demographic variables, baseline comorbidities, virologic factors (HBV DNA levels and HBeAg status), and anti-HBV treatment history. Additionally, we excluded individuals with coinfections and immunocompromising conditions including HIV, HCV, and prior organ transplants to specifically isolate the effect of SLD on CHB-related outcomes. Furthermore, we incorporated the recently proposed definition of SLD (12, 13), which provided a more inclusive study population. This definition acknowledges significant overlap in SLD etiologies and highlights shared pathways involving hepatic lipogenesis, chronic inflammation, and immune dysregulation, all of which contribute to fibrosis, cirrhosis, and HCC (9, 12, 13, 17).

In this study, we found that patients with CHB-SLD had a 17% lower risk of HCC and a 56% reduction in all-cause mortality. These findings are consistent with prior studies suggesting that SLD may have a protective effect against HCC and other adverse outcomes in patients with CHB (10, 18). One proposed mechanism is that SLD inhibits HBV replication by upregulation of Toll-like receptor (TLR) activity, particularly through the TLR4/MyD88 signaling pathway (19, 20). This leads to increased levels of lipopolysaccharides and free fatty acids, which stimulate the production of antiviral cytokines such as interferon-beta and interleukin (IL)-21 (20, 21). These cytokines activate HBV-specific CD4+ and CD8+ T cells, directly inhibiting HBV replication (20, 21). Additionally, pro-inflammatory cytokines such as IL-6, IL-8, and tumor necrosis factor-alpha produced during TLR signaling contribute to immune recovery and viral clearance (21). Other studies suggest that SLD promotes apoptosis of HBV-infected cells through Fas-mediated pathways, resulting in increased HBsAg seroclearance and reduced HBV-DNA levels (22). It has also been suggested that the peroxisome proliferator-activated receptor-gamma coactivator-1 alpha (PGC-1α) pathway, a transcription factor which regulates gluconeogenesis and mitochondrial function, may also play a role (23, 24). Decreased expression of PGC-1α in SLD has been associated with inhibition of HBV replication and altered cellular metabolism (23, 24).

Moreover, we observed that patients with CHB-SLD had lower IRs for detectable serum HBsAg and HBeAg compared with those with CHB-wo-SLD, indicating higher seroclearance rates for both the markers. HBsAg levels correlate highly with covalently closed circular DNA (cccDNA), the episomal HBV reservoir that is responsible for viral persistence in the nucleus of hepatocytes and is a major impediment to HBV cure (25). Loss of HBsAg, with or without seroconversion (i.e., development of anti-HBs antibodies) correlates with reduction in cccDNA (25, 26). Loss of HBsAg represents an important clinical endpoint in the natural history of CHB and signifies functional HBV cure, which is characterized by a lower risk of liver-related complications, including cirrhosis, HCC, and mortality (25, 26). The loss of HBsAg occurs slowly and spontaneously at a frequency of approximately 1% per year; however, this rate can be enhanced by antiviral treatment with nucleos(t)ide analogs or pegylated-interferon alfa-2a (25–27). HBeAg levels, on the other hand, correlate with active viral replication and high HBV DNA levels (28). HBeAg seroclearance, which often precedes or coincides with HBsAg loss, is associated with reduced viral replication, improved liver histology, sustained virological response, and favorable long-term outcomes (28). These results are consistent with the favorable clinical outcomes seen in patients with CHB-SLD compared with those with CHB-wo-SLD.

Despite a lower risk of HCC observed in this study, we found that patients with CHB-SLD had comparable risks of cirrhosis but a paradoxical 1.93-fold higher risk of fibrosis compared to those with CHB-wo-SLD. Similar findings have been reported in large cohort studies, particularly from Asian populations, which have also observed a lower risk of HCC but a higher risk of fibrosis and, in some cases, a higher risk of cirrhosis in CHB-SLD compared to CHB-wo-SLD (29). There are several plausible mechanisms underlying these findings, which can be broadly divided into HBV-specific and SLD-specific effects. Regarding HBV-specific effects, HBV-mediated oncogenesis is known to occur through two distinct mechanisms. The first involves indirect oncogenic effects, responsible for 60-90% of HCC cases, in which chronic HBV infection leads to persistent inflammation, fibrosis, and cirrhosis, which leads to HCC (30–32). The second mechanism involves direct HBV oncogenic effects, which account for 10-40% of HCC cases and are driven by HBV integration into the host genome (30–32). This event leads to insertional mutagenesis, chromosomal instability, and the expression of viral proteins such as HBx and HBs (33–35). HBx, in particular, activates multiple oncogenic signaling pathways, including MAPK, PI3K/AKT, JAK-STAT, and NF-κB, which promote cell proliferation, inhibit apoptosis, and enhance immunosuppression and inflammation (33–36). These effects further activate hepatic stellate cells and Kupffer cells, fostering a profibrogenic liver microenvironment conducive to HCC development (34–36). It is important to also note that the direct oncogenic effects of HBV are independent of cirrhosis, which may explain the comparable risk of HCC between the groups, despite the higher risk of fibrosis observed in individuals with CHB-SLD (34–36).

SLD-specific effects also contribute significantly to the observed paradoxical outcomes and can be best understood by recognizing that the progression from SLD to fibrosis, cirrhosis, and ultimately HCC is influenced by multiple factors along the continuum, which collectively determine overall disease severity and prognosis. For instance, the presence of SLD in individuals with CHB is associated with higher rates of HBsAg loss, as demonstrated by our findings and supported by previous studies (37). Additionally, the SLD phenotype is crucial in determining outcomes. An estimated 20% of patients with CHB-SLD exhibit the steatohepatitis phenotype, which carries a higher risk of progression to fibrosis and cirrhosis compared to simpler or less severe steatosis phenotypes. These less severe forms are characterized by minimal hepatic injury or inflammation and greater responsiveness to pharmacologic or lifestyle interventions (38, 39). Therefore, while the individual risk of fibrosis may be high in patients with SLD, this risk can be offset by several factors, including the presence of a less severe SLD phenotype with a lower likelihood of progression to cirrhosis or HCC. Additionally, these less severe SLD forms are typically more responsive to treatment, which may even halt or reverse fibrosis progression and favorably impact disease outcomes (40). Furthermore, evidence suggests that SLD may contribute to the inhibition of HBV replication and immune recovery in CHB (18, 28). Together, these diverse effects of CHB and SLD plays a critical role in preventing HCC, ESLD events, and other adverse outcomes.

Our study had several limitations. First, it relied on the accuracy of ICD-10 coding for reported clinical data, which can introduce misclassification errors or inconsistencies. Second, the study’s demographic bias towards older, male, and Asian participants may limit the generalizability of the results to other populations. Third, the retrospective study design may have introduced potential biases from missing data and unmeasured confounders, which could have affected the external validity of the study. Fourth, while propensity score matching was used to balance key demographic and clinical characteristics, it also altered some of the risk factor distributions, which may have potentially influenced outcome associations. Specifically, individuals with CHB-SLD were initially older, predominantly male, and had higher BMI, diabetes prevalence, and more frequently of Asian representation, which were subsequently equalized post-matching. This adjustment may have influenced the observed risk factors and outcome associations. Additionally, certain laboratory values such as PTT, albumin, direct bilirubin, and HBV DNA showed greater post-matching differences favoring the CHB-SLD cohort, which may have further accentuated the impact of matching on baseline characteristics. Lastly, the adoption of a broader, inclusive definition of SLD may lead to inconsistencies when compared to other studies that use traditional, more narrowly defined criteria. Despite these limitations, this study had several strengths. To date, this study is one of the largest to provide a comprehensive analysis of clinical outcomes in CHB patients with SLD. Propensity score matching enhances the validity of comparisons between groups. Additionally, the inclusion of a diverse, multisite population across the US increases the relevance and applicability of the study to different clinical settings.

In conclusion, our study demonstrated that, compared to their CHB-wo-SLD counterparts, individuals with CHB-SLD had a lower risk of mortality and adverse outcomes, despite a higher risk of fibrosis. Additionally, the CHB-SLD cohort exhibited significantly lower risks of ESLD events. The CHB-SLD group also showed higher rates of HBsAg and HBeAg seroclearance, which have been associated with improved survival and reduced HCC risk, serving as key endpoints for achieving a functional HBV cure. Importantly, sensitivity analyses of cohorts stratified by cirrhosis status confirmed these findings. Our results highlight the complex interplay between CHB and SLD, suggesting that while SLD may exacerbate certain liver conditions, it simultaneously confers protection against other severe outcomes. This emphasizes the need for a nuanced approach for managing CHB patients with SLD.

## Data Availability

The original contributions presented in the study are included in the article/**Supplementary Material**. Further inquiries can be directed to the corresponding author.
