# Supplementary material for: Impact of hepatic steatosis on mortality, hepatocellular carcinoma, end-stage liver disease and HBsAg seroclearance in chronic hepatitis B: a United States cohort study
Source: Front Immunol. 2025 Apr 2;16:1566925. doi: 10.3389/fimmu.2025.1566925 (PMC12006825; doi:10.3389/fimmu.2025.1566925)
Supplement: Supplementary file 1 [file Table1.docx]

Supplementary Material

**Supplemental File 1.** Cohort inclusion and exclusion criteria and coding systems

| **Baseline characteristics** | Coding system | Code(s) |
| --- | --- | --- |
| Chronic Hepatitis B | ICD-10 | B18.1, B18.0 |
| Steatotic liver disease (fatty liver) | ICD-10 | K70.0, K75.81, K76.0 |
| Diseases of liver | ICD-10 | K70-K77 |
| Ischemic heart diseases | ICD-10 | I20-I25 |
| Hypertensive diseases | ICD-10 | I10-I16 |
| Heart failure | ICD-10 | I50 |
| Diabetes mellitus | ICD-10 | E08-E13 |
| Overweight or obesity | ICD-10 | E66 |
| Chronic lower respiratory diseases | ICD-10 | J40-J47 |
| Chronic kidney disease | ICD-10 | N18 |
| Neoplasms | ICD-10 | C00-D49 |
| HIV disease | ICD-10 | B20 |
| Chronic HCV | ICD-10 | B18.2 |
| Nicotine dependence | ICD-10 | F17.2 |
| Alcohol related disorders | ICD-10 | F10 |
| **Outcomes** |  |  |
| Deceased (Mortality) | No specific code |  |
| Hepatocellular carcinoma | ICD-10 | C22.0, C22.8, C22.7 |
| Fibrosis | ICD-10 | K74.0 |
| Cirrhosis | ICD-10 | K74.60, K74.69, K74.0, K70.31, K70.30, K70.3, K71.7 |
| Ascites | ICD-10 | R18, R18.8 |
| Spontaneous Bacterial Peritonitis (SBP) | ICD-10 | K65.2 |
| Variceal Bleeding | ICD-10 | I85.01 |
| Hepatic Encephalopathy | ICD-10 | K76.82 |
| Hepatorenal Syndrome | ICD-10 | K76.7, K91.83 |
| HBsAg | CPT | 87340, 87341, 87340, 80074, 87467 |
| HBeAg | CPT | 87350 |

**Supplementary File 2.** Primary analysis of unmatched cohorts

| Outcomes | Overall | CHB-SLD | CHB-wo-SLD | HR (95% CI) | p-Value |
| --- | --- | --- | --- | --- | --- |
|  |  |  |  |  |  |
| General |  |  |  |  |  |
| Mortality (all-cause) | 117,047 | 855 (5.8%) | 8,732 (8.5%) | 0.64 (0.60-0.69) | **<0.001** |
| Hepatic |  |  |  |  |  |
| Fibrosis | 112,345 | 917 (6.9%) | 2,222 (2.2%) | 3.01 (2.79-3.25) | **<0.001** |
| Cirrhosis | 107,115 | 920 (7.3%) | 4,420 (4.7%) | 1.51 (1.41-1.62) | **<0.001** |
| Hepatocellular carcinoma | 110,751 | 399 (2.8%) | 2,099 (2.2%) | 1.26 (1.33-1.41) | **<0.001** |
| Ascites | 113,896 | 402 (2.8%) | 2,689 (2.7%) | 1.00 (0.90-1.11) | 0.977 |
| End-stage Liver Disease |  |  |  |  |  |
| Spontaneous bacterial peritonitis | 117,182 | 59 (0.4%) | 482 (0.5%) | 0.80 (0.61-1.05) | 0.103 |
| Variceal bleeding | 115,482 | 287 (2.0%) | 1,493 (1.5%) | 1.27(1.12-1.44) | **0.005** |
| Hepatic encephalopathy | 117,098 | 111 (0.8%) | 387 (0.4%) | 1.91 (1.55-2.36) | **<0.001** |
| Hepatorenal syndrome | 117,173 | 69 (0.5%) | 485 (0.5%) | 0.92 (0.72-1.19) | 0.526 |
| Virologic |  |  |  |  |  |
| HBsAg | 109,415 | 994 (9.9%) | 9,159 (10.8%) | 0.87 (0.81-0.92) | **<0.001** |
| HBeAg | 94,791 | 406 (3.0%) | 4,459 (4.7%) | 0.61 (0.55-0.67) | **<0.001** |

CI, confidence interval; CHB-SLD, patients with chronic hepatitis B and steatotic liver disease; CHB-wo-SLD, patients with chronic hepatitis B without steatotic liver disease; HBeAg, hepatitis B e antigen; HBsAg, hepatitis B surface antigen; HR, hazard ratio

**Supplementary File 3.** Sensitivity analysis of unmatched cohorts (minus HIV and HCV)

| Outcomes | No Cirrhosis | | | | | Cirrhosis | | | | |
| --- | --- | --- | --- | --- | --- | --- | --- | --- | --- | --- |
|  | **Overall** | **CHB-SLD** | **CHB-**  **wo-SLD** | **HR (95% CI)** | **p-Value** | **Overall** | **CHB-SLD** | **CHB-**  **wo-SLD** | **HR (95%**  **CI)** | **p-Value** |
| General |  |  |  |  |  |  |  |  |  |  |
| Mortality (all-cause) | 106,809 | 412 (3.4%) | 5,823 (6.2%) | 0.53 (0.48-0.59) | **<0.001** | 16,143 | 464 (14.5%) | 3,222 (24.9%) | 0.51 (0.46-0.56) | **<0.001** |
| Hepatic |  |  |  |  |  |  |  |  |  |  |
| Fibrosis | 106,298 | 475 (4.0%) | 833 (0.9%) | 4.63 (4.13-5.18) | **<0.001** | 10,362 | 180 (9.8%) | 332 (3.9%) | 2.20 (1.84-2.64) | **<0.001** |
| Hepatocellular carcinoma | 103,969 | 194 (1.6%) | 982 (1.1%) | 1.52 (1.30-1.77) | **<0.001** | 12,198 | 174 (6.1%) | 862 (9.2%) | 0.64 (0.54-0.76) | **<0.001** |
| End-stage Liver Disease |  |  |  |  |  |  |  |  |  |  |
| Ascites | 105,966 | 129 (1.1%) | 1,016 (1.1%) | 0.96 (0.80-1.15) | 0.662 | 13,318 | 235 (8.6%) | 1,329 (12.6%) | 0.61 (0.53-0.70) | **<0.001** |
| Spontaneous bacterial peritonitis | 107,083 | 10 (0.1%) | 83 (0.1%) | 0.44 (0.18-1.10) | 0.079 | 15,996 | 53  (1.7%) | 371 (2.9%) | 0.51 (0.38-0.68) | **<0.001** |
| Variceal bleeding | 106,954 | 18  (0.1%) | 118 (0.1%) | 1.15 (0.70-1.90) | 0.572 | 14,139 | 236 (8.5%) | 1,180 (10.4%) | 0.71 (0.61-0.81) | **<0.001** |
| Hepatic encephalopathy | 107,076 | 10 (0.1%) | 31 (0.1%) | 1.94 (0.89-4.22) | 0.089 | 15,921 | 102 (3.3%) | 342 (2.7%) | 1.07 (0.86-1.34) | 0.542 |
| Hepatorenal syndrome | 107,060 | 10 (0.1%) | 69 (0.1%) | 0.32 (0.10-1.03) | 0.098 | 16,015 | 64 (2.0%) | 377 (2.9%) | 0.60 (0.46-0.79) | **<0.001** |
| Virologic |  |  |  |  |  |  |  |  |  |  |
| HBsAg | 100,664 | 751 (9.0%) | 8,099 (10.5%) | 0.83 (0.77-0.89) | **<0.001** | 14,397 | 256 (15.3%) | 1,486 (17.6%) | 0.80 (0.70-0.92) | **0.001** |
| HBeAg | 85,459 | 313 (2.8%) | 3,653 (4.1%) | 0.65 (0.58-0.73) | **<0.001** | 10,114 | 90  (3.1%) | 728 (6.3%) | 0.44 (0.36-0.550 | **<0.001** |

CI, confidence interval; CHB-SLD, patients with chronic hepatitis B and steatotic liver disease; CHB-wo-SLD, patients with chronic hepatitis B without steatotic liver disease; HBeAg, hepatitis B e antigen; HBsAg, hepatitis B surface antigen; HR, hazard ratio

##

**
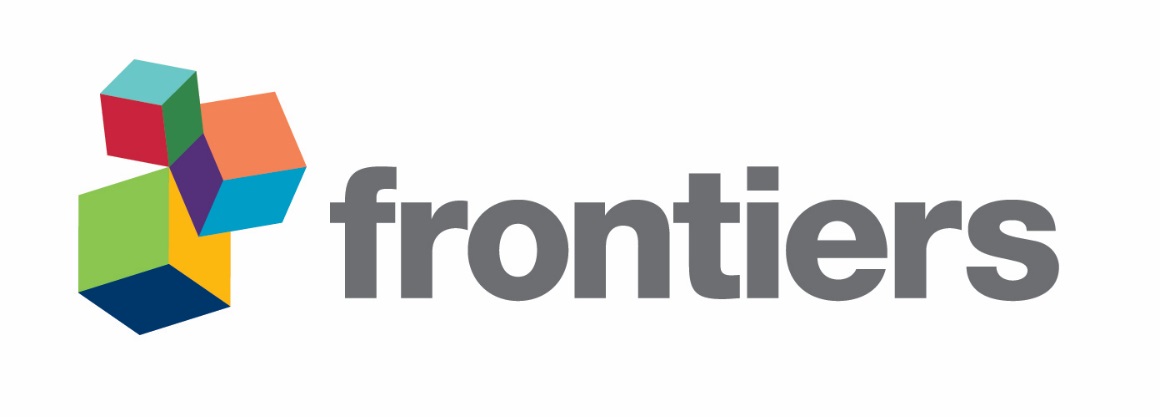
**
